# Star Centroiding Based on Fast Gaussian Fitting for Star Sensors

**DOI:** 10.3390/s18092836

**Published:** 2018-08-28

**Authors:** Xiaowei Wan, Gangyi Wang, Xinguo Wei, Jian Li, Guangjun Zhang

**Affiliations:** School of Instrumentation Science and Opto-electronics Engineering, Beihang University, 37 Xueyuan Rd., Haidian District, Beijing 100191, China; sengesky@buaa.edu.cn (X.W.); wxg@buaa.edu.cn (X.W.); lijian_0355@163.com (J.L.); gjzhang@buaa.edu.cn (G.Z.)

**Keywords:** star sensor, star centroiding, Gaussian fitting, real-time

## Abstract

The most accurate star centroiding method for star sensors is the Gaussian fitting (GF) algorithm, because the intensity distribution of a star spot conforms to the Gaussian function, but the computational complexity of GF is too high for real-time applications. In this paper, we develop the fast Gaussian fitting method (FGF), which approximates the solution of the GF in a closed-form, thus significantly speeding up the GF algorithm. Based on the fast Gaussian fitting method, a novel star centroiding algorithm is proposed, which sequentially performs the FGF twice to calculate the star centroid: the first FGF step roughly calculates the Gaussian parameters of a star spot and the noise intensity of each pixel; subsequently the second FGF accurately calculates the star centroid utilizing the noise intensity provided in the first step. In this way, the proposed algorithm achieves both high accuracy and high efficiency. Both simulated star images and star sensor images are used to verify the performance of the algorithm. Experimental results show that the accuracy of the proposed algorithm is almost the same as the GF algorithm, higher than most existing centroiding algorithms, meanwhile, the proposed algorithm is about 15 times faster than the GF algorithm, making it suitable for real-time applications.

## 1. Introduction

Star sensors are indispensable components of spacecraft attitude measurement systems. They capture images of stars with an imaging chip, and determine the three-axis attitude according to the location of stars in the field of view (FOV) [1]. In recent years, with the rapid development of space exploration [2,3,4], higher attitude accuracy is being more and more required, making it imperative to improve the accuracy of star sensors. Many factors can affect the accuracy of a star sensor, including the size of FOV, sensitivity of the imaging chip, distortion of the lens, accuracy of star centroiding, etc. In this work, we focus on improving the star centroiding accuracy.

A typical method to get an accurate star centroid is to make the star sensor slightly defocused, making the size of a star spot not smaller than 3 × 3 pixels. The intensity of a de-focused star spot conforms to the “Airy disk”, which can be approximated by the Gaussian function [5]. The task of a star centroiding algorithm is to estimate the centroid of the star spot.

Many algorithms have been developed for star centroiding, which can be divided into two classes: the gray scale centroid methods and the fitting methods. The gray scale centroid methods estimate the star centroid by calculating the center of gravity of the star spot, among which the most commonly used algorithm is the center of gravity algorithm (CG) [6]. The formula of the CG is:

(1) $$\left(x_{c},y_{c}\right)=\left(\frac{\sum_{i}I_{i}x_{i}}{\sum_{i}I_{i}},\frac{\sum_{i}I_{i}y_{i}}{\sum_{i}I_{i}}\right)$$

where i represents the *i*-th pixel; $x_{c}$ and $y_{c}$ represent the centroid of the star spot; $I_{i}$ represents the gray value of pixel *i*; $x_{i}$ and $y_{i}$ represent the coordinates of pixel *i*. The CG is simple and effective, but the accuracy of this algorithm is restricted by the S-curve error, which is introduced by discrete sampling of the star image [7].

A typical way to improve the accuracy of the CG is to insert a set of weight values in the formula of the CG, which is named the weighted center of gravity algorithm (WCG) [8]. The formula of the WCG is:

(2) $$\left(x_{c},y_{c}\right)=\left(\frac{\sum_{i}W_{i}I_{i}x_{i}}{\sum_{i}W_{i}I_{i}},\frac{\sum_{i}W_{i}I_{i}y_{i}}{\sum_{i}W_{i}I_{i}}\right)$$

where $W_{i}$ is the weight of the *i*-th pixel.

Theoretically, the weight values should be assigned by the Gaussian function [9], however, those values could not be easily obtained. In the squared gray centroid method [10], the weight $W_{i}$ is approximated by the intensity $I_{i}$ based on the fact that the intensity of a star spot conforms to the Gaussian function. The accuracy of this algorithm is significantly improved when the radius of the star spot is large enough, however, the accuracy decreases rapidly when the radius gets smaller.

The fitting methods estimate the star centroid by fitting the intensity of a star spot to the Gaussian function. As the star spot conforms to a 2-dimensional Gaussian function, the Gaussian fitting algorithm (GF) can in theory achieve the highest star centroiding accuracy [11,12,13]. However, since solving the Gaussian parameters is a nonlinear optimization problem, which is inevitably a multiple-step iteration process, the algorithm is quite sensitive to the initial parameters and is time consuming in practice. To address the shortcomings of the GF, the Gaussian analysis algorithm (GA) is proposed in [14], where it is assumed that there is no intensity noise in the star images and a closed-form solution of the Gaussian parameters is derived, significantly speeding up the GF, but the algorithm is sensitive to intensity noise. Another way to improve the GF is to divide the Gaussian fitting into multiple 1-dimensional Gaussian fittings [15]. These algorithms speed up the GF to some extent at the cost of decreasing the accuracy.

Besides the above two classes of star centroiding methods, some other new star centroiding methods were proposed recently. For instance, Flewelling in [16] presented a star centroiding method with information theoretic weighting. Although the method achieves quite high accuracy, the high computational complexity makes it unsuitable for real-time applications.

In this paper, we propose a star centroiding algorithm based on the fitting method. By transforming the objective function of the GF to a simpler form, the proposed algorithm can solve the Gaussian parameters without iteration, making it more efficient than the GF. On the other hand, the algorithm performs similarly as the GF in accuracy and noise robustness.

This paper is organized as follows: in Section 2, we develop an algorithm to quickly solve the Gaussian parameters and propose a novel star centroiding algorithm. In Section 3, experiments are designed to verify the performance of the algorithm.

## 2. Method Description

### 2.1. Imaging Characteristics of the Star Sensor

A star sensor is an imaging system that uses an optical system to image stars onto the array’s photosensitive area. Its output is a series of numerical values that represent the scene intensity at a series of discrete locations [17]. The imaging model of star sensor can be formulated as:

(3) $$I_{i}=S_{i}+N_{i}$$

where $I_{i}$, $S_{i}$ and $N_{i}$ represent the observed, source and noise intensity of pixel i, respectively.

As described above, the source intensity of each pixel in the star spot can be expressed by a Gaussian function:

(4) $$S\left(x_{i},y_{i}|v\right)=A\exp\left(-\frac{{\left(x_{i}-x_{c}\right)}^{2}}{2\sigma_{x}^{2}}-\frac{{\left(y_{i}-y_{c}\right)}^{2}}{2\sigma_{y}^{2}}\right)$$

where $x_{i}$ and $y_{i}$ represent the coordinates of pixel *i*; v represents the Gaussian parameters $\left(A,\;x_{c},\;y_{c},\;\sigma_{x},\;\sigma_{y}\right)$; A represents the brightness level of the star; $x_{c}$ and $y_{c}$ represent the centroid of the star; $\sigma_{x}$ and $\sigma_{y}$ represent the standard deviation of the function.

### 2.2. Characteristics of Gaussian Fitting

As the most accurate star centroiding method, the GF estimates the star centroid by fitting the observed intensity of a star to the Gaussian function. The objective function of the GF is:

(5) $$Z=\arg\underset{v}{\min}\underset{i\in U}{\sum }{\left[z_{i}\right]}^{2}$$

where $z_{i}=I\left(x_{i},y_{i}\right)-S\left(x_{i},y_{i}|v\right)$, and U is a set formed by the pixels in the star spot.

According to the theory of error, the star centroid estimated with Equation (5) is the optimum estimation in the maximum likelihood sense when the noise of each pixel conforms to a Gaussian distribution, which can be usually satisfied for star images. However, since Equation (5) is a nonlinear least squares problem, complex algorithms such as the Levenberg-Marquardt algorithm [18] or the trust-region-reflective algorithm [19] are needed to solve it. These algorithms usually start from an initial guess of the parameters and approach the optimal solution in a multiple iteration procedure [20], which is rather time-consuming. On the other hand, improper initial guess of the parameters may make the algorithms converge to local optimal parameters, leading to instability in the results [21].

### 2.3. Fast Gaussian Fitting

To overcome the shortcomings of the GF and preserve its accuracy, we develop the fast Gaussian fitting method in this section.

Modify the objective function of the GF by performing logarithm operation on both I and S in Equation (5) as follows:

(6) $$G=\arg\underset{v}{\min}\underset{i\in U}{\sum }{\left[g_{i}\right]}^{2}$$

where $g_{i}=\ln I\left(x_{i},y_{i}\right)-\ln S\left(x_{i},y_{i}|v\right)$.

By substituting Equation (4) into Equation (6), it is easy to see that the optimization problem in Equation (6) is a linear least squares problem, which can be solved in a closed-form very efficiently. If Equation (6) shares the same solution with Equation (5) then it is a good acceleration method for the GF.

When there is no noise in images, i.e., N=0, it is clear that Equation (6) is equivalent with Equation (5). However, in real star images, noise is always present and cannot be completely removed, i.e., N≠0. Therefore, the solution of Equation (6) is probably different from that of Equation (5).

By substituting Equation (3) into Equation (5) and Equation (6), the two objective functions can be converted to:

(7) $$Z=\arg\underset{v}{\min}\underset{i\in U}{\sum }{\left[N_{i}\right]}^{2}$$

(8) $$G=\arg\underset{v}{\min}\underset{i\in U}{\sum }{\left[w_{i}N_{i}\right]}^{2}$$

where:

(9) $$w_{i}=\frac{1}{N_{i}}\ln\left(1+\frac{N_{i}}{S_{i}}\right)$$

**Figure 1:**
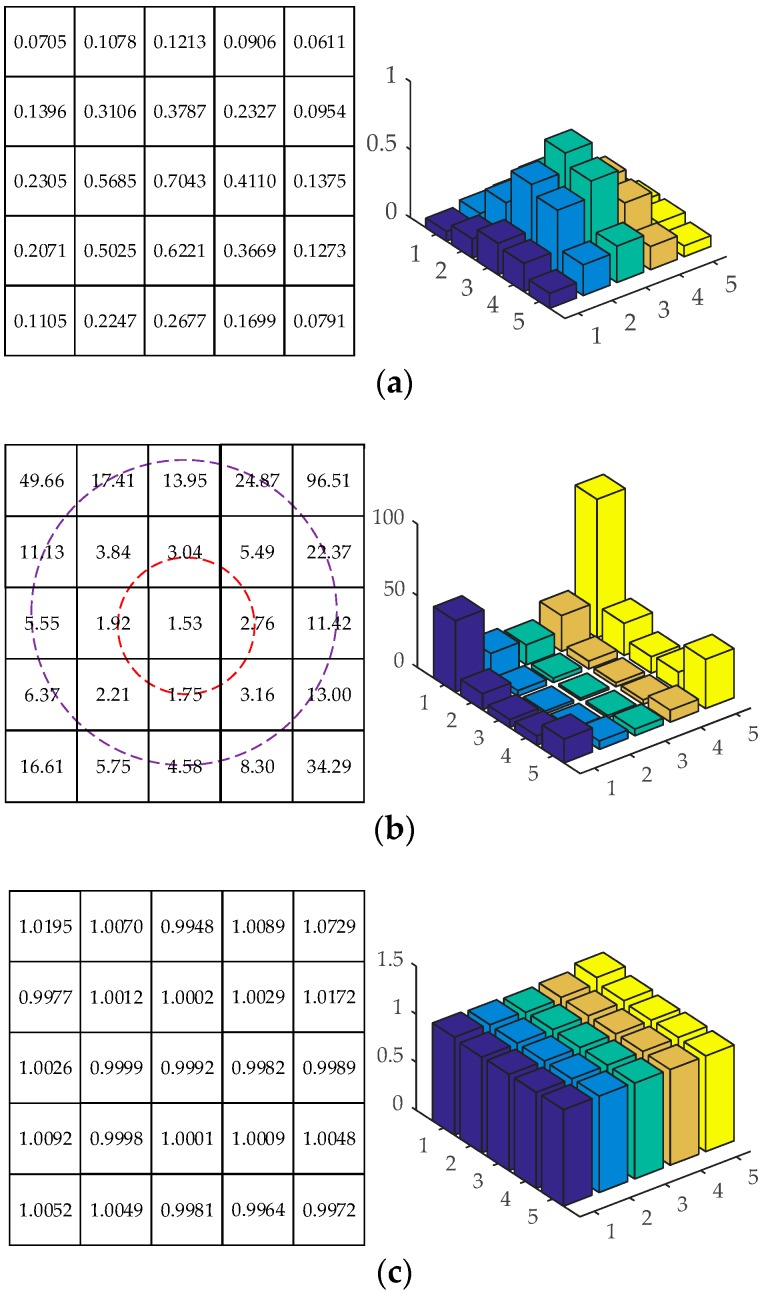
The intensity I, weights w and φ(r) of a star spot. (**a**) is the intensity of the star spot; (**b**) is the weights w in Equation (8) of the star spot; (**c**) is the weights φ(r) in Equation (12) of the star spot. The intensity of the pixels in the center region is greater than that of the pixels in the edge region in (**a**). The weights of pixels in the center region are much less than that of pixels in the edge region in (**b**). All the values in (**c**) are very close to 1.

This means Equation (8) can be seen as a weighted GF, and the weight of each pixel is determined by the intensity of source and noise of the pixel. For a certain noise intensity, a lower source intensity leads to a higher weight, which makes Equation (8) tend to be more sensitive to the pixels with lower source intensity. We illustrate this phenomenon with a simulated star spot in Figure 1. The intensity and weight of each pixel are shown in Figure 1a,b, respectively. Compared with the pixels in the center region, the pixels located at the edge of the star spot are much darker, but their weights are multiple times larger, which means the solution of Equation (8) is mainly determined by the edge pixels. Since the intensity of the edge pixels is easier to be influenced by noise, the center estimated with Equation (6) is more sensitive to noise than the GF, which is not what we expect.

To address this problem, we need to eliminate the nonuniformity of the weights. $g_{i}$ in Equation (6) can be transformed into:

(10) $$g_{i}=\ln I_{i}-\ln S_{i}=N_{i}\frac{1}{I_{i}}\left(1+\frac{S_{i}}{N_{i}}\right)\ln\left(1+\frac{N_{i}}{S_{i}}\right)=N_{i}\frac{1}{I_{i}}\varphi \left(\frac{S_{i}}{N_{i}}\right)$$

where $\varphi \left(\frac{S_{i}}{N_{i}}\right)=\left(1+\frac{S_{i}}{N_{i}}\right)\ln\left(1+\frac{N_{i}}{S_{i}}\right)$.

Let $r_{i}=\frac{S_{i}}{N_{i}}$, and define $\left|r_{i}\right|$ as the signal-to-noise ratio (SNR) of the *i*-th pixel. $\varphi \left(\frac{S_{i}}{N_{i}}\right)$ can be transformed into:

(11) $$\varphi \left(r_{i}\right)=\left(1+r_{i}\right)\ln\left(1+\frac{1}{r_{i}}\right)$$

**Figure 2:**
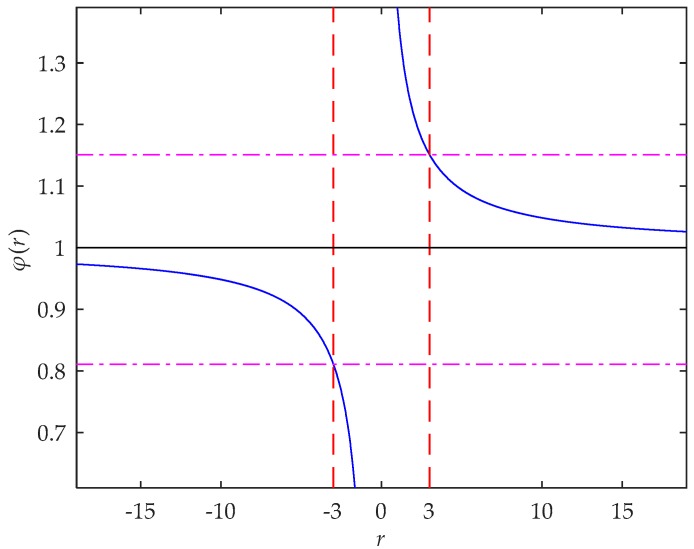
The curve of φ(r). The value of φ(r) converges to 1 with the increase of SNR. When r is equal to 3, the value of φ(r) is equal to 1.1507. When r is equal to −3, the value of φ(r) is equal to 0.8109.

When $r_{i}$ of pixel i ranges from −1 to 1, i.e., SNR of pixel i is less than 1, the pixel is submerged in the noise and is usually excluded from the star spot area, thus there is no need to analyze this case. When SNR of pixel i is greater than 1, the value of φ(r) converges to 1 with the increase of the SNR, as shown in Figure 2.

Considering the values of φ(r) of pixels in the star spot are close to 1, we modify Equation (6) by adding weight $I_{i}$ in the equation as follows:

(12) $$H=\arg\underset{v}{\min}\underset{i\in U}{\sum }{\left[I_{i}(\ln I_{i}-\ln S_{i})\right]}^{2}=\arg\underset{v}{\min}\underset{i\in U}{\sum }{\left[\varphi \left(r_{i}\right)N_{i}\right]}^{2}$$

where U is a set formed by the pixels whose φ(r) are close to 1.

Equation (12) can also be seen as a weighted GF with the weight of each pixel determined by $\varphi \left(r_{i}\right)$. Figure 1c illustrates the values of φ(r) for the star spot in Figure 1a. Different from the weights in Figure 1b, all the values of φ(r) in Figure 1c are very close to 1, indicating that the solution of Equation (12) can be approximately equal to Equation (5), and is more robust to noise than that of Equation (6). 

It can be seen from Figure 2, when the SNR of a pixel is greater than a certain value, the value of φ(r) can be limited in any given neighborhood of 1. For example, when SNR>3, φ(r)∈(0.8109,1.1507); when SNR > 10, φ(r)∈(0.9482,1.0484). Therefore, we can select the pixels with SNR greater than a certain value to ensure that φ(r) of these pixels is close enough to 1.

Moreover, Equation (12) is also a linear least squares problem, which can be solved efficiently in closed-form, making it a good acceleration method for the GF. We name the method as the fast Gaussian fitting method.

The process of finding the optimal solution of Equation (12) is described below. By substituting Equation (4) to Equation (12), Equation (12) is converted into:

(13) $$H=\arg\underset{v}{\min}\underset{i\in U}{\sum }{\left(\frac{I_{i}x_{i}{}^{2}}{2\sigma_{x}^{2}}+\frac{I_{i}y_{i}{}^{2}}{2\sigma_{y}^{2}}-\frac{I_{i}x_{c}x_{i}}{\sigma_{x}^{2}}-\frac{I_{i}y_{c}y_{i}}{\sigma_{y}^{2}}+\frac{I_{i}x_{c}^{2}}{2\sigma_{x}^{2}}+\frac{I_{i}y_{c}^{2}}{2\sigma_{y}^{2}}+I_{i}\ln I_{i}-I_{i}\ln A\right)}^{2}$$

For the convenience of calculations, m, n, p, q and k are defined follows:

(14) $$\{\begin{array}{c} m=\frac{1}{2\sigma_{x}^{2}}\\ n=\frac{1}{2\sigma_{y}^{2}}\\ p=-\frac{x_{c}}{\sigma_{x}^{2}}\\ q=-\frac{y_{c}}{\sigma_{y}^{2}}\\ k=\frac{x_{c}^{2}}{2\sigma_{x}^{2}}+\frac{y_{c}^{2}}{2\sigma_{y}^{2}}-\ln A \end{array}$$

By substituting Equation (14) to Equation (13), the objective function is converted into:

(15) $$H=\arg\underset{m,n,p,q,k}{\min}\underset{i\in U}{\sum }{\left(mI_{i}x_{i}{}^{2}+nI_{i}y_{i}{}^{2}+pI_{i}x_{i}+qI_{i}y_{i}+kI_{i}+I_{i}\ln I_{i}\right)}^{2}=\arg\underset{m,n,p,q,k}{\min}\underset{i\in U}{\sum }h_{i}^{2}$$

For the parameters m, n, p, q and k, Equation (15) is a least squares problem. The solution can be found by solving the following equation:

(16) $$\{\begin{array}{c} \frac{\partial H}{\partial m}=\underset{i\in U}{\sum }2I_{i}x_{i}{}^{2}h_{i}=0\\ \frac{\partial H}{\partial n}=\underset{i\in U}{\sum }2I_{i}y_{i}{}^{2}h_{i}=0\\ \frac{\partial H}{\partial p}=\underset{i\in U}{\sum }2I_{i}x_{i}h_{i}=0\\ \frac{\partial H}{\partial q}=\underset{i\in U}{\sum }2I_{i}y_{i}h_{i}=0\\ \frac{\partial H}{\partial k}=\underset{i\in U}{\sum }2I_{i}h_{i}=0 \end{array}$$

By using the derivative operation, we can find the critical point of each parameter, and solve the optimal parameters $\bar{m}$, $\bar{n}$, $\bar{p}$, $\bar{q}$ and $\bar{k}$. The corresponding parameters $\left(A,\;x_{c},\;y_{c},\;\sigma_{x},\;\sigma_{y}\right)$ can be solved as follows:

(17) $$\{\begin{array}{c} x_{c}=-\frac{\bar{p}}{2\bar{m}}\\ y_{c}=-\frac{\bar{q}}{2\bar{n}}\\ \sigma_{x}=\frac{1}{\sqrt{2\bar{m}}}\\ \sigma_{y}=\frac{1}{\sqrt{2\bar{n}}}\\ A=\exp\left(\frac{{\bar{p}}^{2}}{4\bar{m}}+\frac{{\bar{q}}^{2}}{4\bar{n}}-\bar{k}\right) \end{array}$$

### 2.4. Star Centroiding Based on Fast Gaussian Fitting

As described above, the objective function of the fast Gaussian fitting method can be approximately equal to that of the GF under the precondition: φ(r) of each pixel involved in the calculation is close enough to 1. In star images, due to the uncertainty of the noise and star intensity, not all pixels satisfy the precondition, and it does not provide how to select the pixels with SNR greater than a certain value. Therefore, the fast Gaussian fitting method cannot be used as a complete star centroiding method. In this section, we design a method to select the pixels that satisfy the precondition by thresholding the SNR of pixels, and propose a star centroiding algorithm based on the fast Gaussian fitting method.

**Figure 3:**
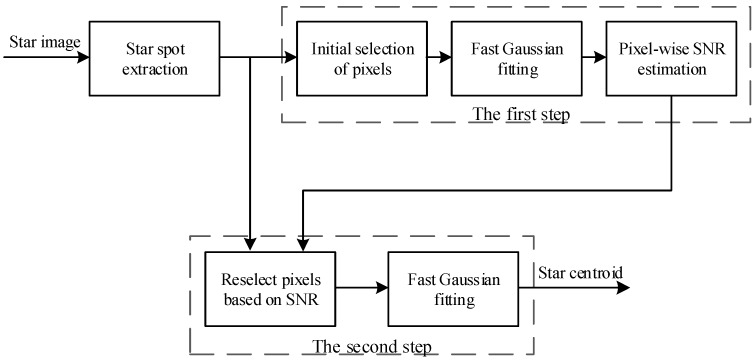
The flow diagram of the FGF.

The proposed star centroiding algorithm is summarized as follows: when a star spot is extracted, two steps are designed to solve the star spot centroid. In the first step, we get a rough solution of the Gaussian parameters of the star spot with the FGF, then use the solution to estimate the noise and SNR of each pixel in the star spot. In this way, the SNR of each pixel in the star spot is obtained, and the pixels that satisfy the precondition in the FGF can be found by thresholding the SNR. In the second step, we select all the pixels that satisfy the precondition and use the FGF to calculate the star centroid. Compared with the FGF in the first step, more pixels are involved in the second step, and the solution is more accurate. The working procedure is shown in Figure 3.

(1) Star spot extraction: When a star image is obtained, the connected components are extracted to locate the star spots in the image. The number of pixels in each star spot should be no less than 5, otherwise, the star spot will be considered as a false star and removed. In addition, the pixels with full saturated gray value in the star spot are excluded.
(2) The first step: The task of this step is to estimate the SNR of each pixel. The detailed process is as follows:
  (i) Initial selection of pixels: Although the noise of each pixel is unknown, the pixels with larger gray values usually have higher SNR, thus their corresponding φ(r) are closer to 1. According to this idea, the five pixels with the maximum gray values in the star spot are selected as the initial pixels of the set U.
  (ii) Fast Gaussian fitting: The fast Gaussian fitting method described above is adopted to find the solution of the Gaussian parameters $\left(A^{\prime },\;x_{c}^{\prime },\;y_{c}^{\prime },\;\sigma_{x}^{\prime },\;\sigma_{y}^{\prime }\right)$. With these parameters, we can establish the star intensity function, which is:
    (18) $${\hat{S}}_{i}=A^{\prime }\exp\left(-\frac{{\left(x_{i}-x_{c}^{\prime }\right)}^{2}}{2\sigma_{x}^{\prime }{}^{2}}-\frac{{\left(y_{i}-y_{c}^{\prime }\right)}^{2}}{2\sigma_{y}^{\prime }{}^{2}}\right)$$
  (iii) Pixel-wise SNR estimation: According to Equation (3) and Equation (18), the noise intensity of each pixel in the star spot can be calculated by Equation (19), and the SNR of each pixel can be estimated by Equation (20):
    (19) $${\hat{N}}_{i}={\hat{I}}_{i}-{\hat{S}}_{i}=I\left(x_{i},y_{i}\right)-A^{\prime }\exp\left(-\frac{{\left(x_{i}-x_{c}^{\prime }\right)}^{2}}{2\sigma_{x}^{\prime }{}^{2}}-\frac{{\left(y_{i}-y_{c}^{\prime }\right)}^{2}}{2\sigma_{y}^{\prime }{}^{2}}\right)$$
    (20) $${\mathrm{SNR}}_{i}=|\frac{{\hat{S}}_{i}}{{\hat{N}}_{i}}|$$
(3) The second step: Since the SNR of each pixel has been estimated in the previous step, we can solve the star centroid as follows:
  (i) Reselect pixels based on SNR: In this step, we will update the pixels in the set U by thresholding the SNR. The pixels in the star spot are filtered by judging whether the SNR of the pixel is greater than a threshold and those pixels with SNR greater than the threshold are collected to form the set U. T is used as the value of the threshold:
    (21) $$U=\{i,{\;\mathrm{SNR}}_{i}>T\}$$
  (ii) Fast Gaussian fitting: The fast Gaussian fitting method is adopted to find the optimal solution of the Gaussian parameters again. Comparing with the first step in the noise estimation step, more pixels are involved in this step, and the results will be more accurate.

## 3. Results and Discussion

To evaluate the performance of the proposed algorithm, we test the algorithm on a set of simulated star images with different characteristics, and compare with the existing star centroiding methods. In the following sub-sections, we describe generation of the simulated star images, selection of parameters, and experiments in detail.

### 3.1. Star Images Generation

In this work, the star images are generated to simulate the images obtained by a star sensor. Each star image is of 31 × 31 pixels, and includes only one star spot.

In the simulated star images, each pixel is generated according to Equation (4), as follows:

(22) $$f_{i}=A\exp\left(-\frac{{\left(x_{i}-x_{c}\right)}^{2}}{2\sigma_{x}^{2}}-\frac{{\left(y_{i}-y_{c}\right)}^{2}}{2\sigma_{y}^{2}}\right)+N_{i}$$

where $x_{i}$ and $y_{i}$ represent the i-th pixel in the image; $x_{c}$ and $y_{c}$ represent the centroid of the star spot in pixels; $\sigma_{x}=\sigma_{y}=\sigma_{r}$ represents the standard deviation of the generated function of the star spot, and is defined as the Gaussian radius here; A represents the brightness level here; $N_{i}$ represents the noise intensity of pixel i. The value of $N_{i}$ is a random value which obeys Gaussian-distributed with 0 mean and $\sigma_{g}$ standard deviation.

### 3.2. Parameter Selection

The threshold T in the fast Gaussian fitting method is a key parameter of the proposed algorithm. We select the value of the parameter as follows:

The threshold T is used to select the pixels involved in the star centroiding step. Two aspects should be taken into account to select the value of T:

(i) The number of pixels in set U must be no less than 5;
(ii) φ(r) of each pixel selected should be close enough to 1.

If the value of T is very large, φ(r) of each pixel in set U is closer to 1, but the number of pixels in U is reduced. On the other hand, if the value of T is very small, the number of the pixels in U increases, but φ(r) of some pixels in U may be far away from 1, which can reduce the accuracy of the fast Gaussian fitting method. Considering the diversity of the star images, we set T=3.

### 3.3. Accuracy Experiments

The proposed algorithm is verified by a series of star images with different centroid, Gaussian radius, noise level and brightness level. The accuracy of the algorithm is analyzed by the star centroiding error, which is defined as the Euclidean distance between the calculated centroid $\left({\hat{x}}_{c},{\hat{y}}_{c}\right)$ and the true centroid $\left(x_{c},y_{c}\right)$:

(23) $$e=\sqrt{{\left({\hat{x}}_{c}-x_{c}\right)}^{2}+{\left({\hat{y}}_{c}-y_{c}\right)}^{2}}$$

Several existing star centroiding methods are adopted to compare with the proposed algorithm. The center of gravity algorithm (CG) is considered to be the least time-consuming method, and the squared gray centroid method is the most common weighted center of gravity algorithm (WCG), which is an improvement of the CG. The Gaussian fitting algorithm (GF) is the most accuracy method in theory, and the Gaussian analytic method (GA) is the latest improved Gaussian Fitting algorithm, which is claimed to be the most accurate and efficient algorithm when the size of the star spot radius is not bigger than 5×5. All of the experiments are carried on the MATLAB 2016b software platform running on an Intel Core i5-7500T 2.7 GHz processor.

#### 3.3.1. Experiment with the Star Spot at Different Locations

**Figure 4:**
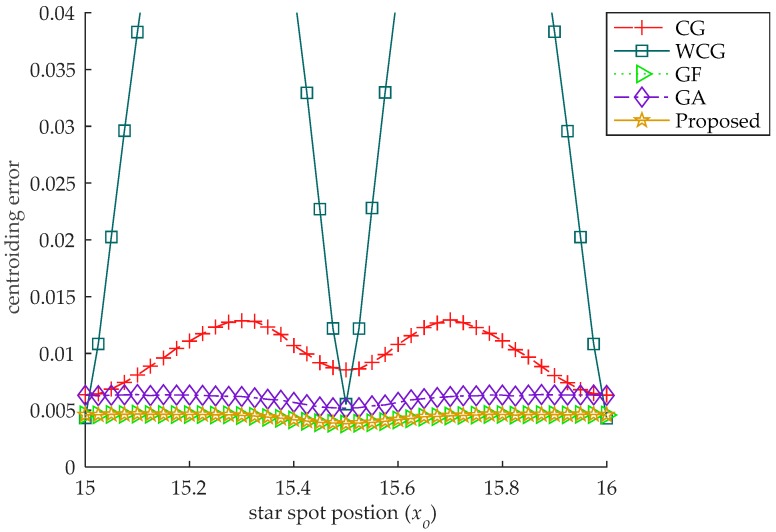
The centroiding error with the star spot at different locations. There is a sine curve in the pixel for the CG and WCG, and the error of the WCG is greater than that of the CG.

In this experiment, the accuracy of the algorithm is verified by moving the centroid of the star spot from one side of a pixel to the other. Considering the symmetry of the star spot, the star spot is set to move along the x-axis at the center of the pixel, which is located at the center of the image, i.e., $x_{o}$ ranges from 15 to 16, and $y_{o}=15.5$. The Gaussian radius of the star spot is 0.5, the brightness level is 0.7, and the standard deviation of the Gaussian noise is 0.001, which is equivalent to σ=4.096 for a 12-bit AD CMOS. For each location of the star spot, 10,000 images are generated, and the mean centroiding error of the 10,000 images is used as the centroiding error. The error curves are shown in Figure 4.

There are three special points (15, 15.5), (15.5, 15.5) and (16, 15.5) in this experiment. When the star spot is located in any of the three points, the centroiding error of the CG gets its local minimum value. When the star spot moves along the x-axis in the pixel, the centroiding error curve of the CG is a sine-like curve. There is a similar phenomenon for the WCG, whose error is greater than that of the CG. There is no obvious such phenomenon for the GF, the GA and the proposed star centroiding algorithm, whose error curves are stable when the star spot moves from one side to another.

This phenomenon is caused by the S-curve error, which is an artefact of the sampling theorem due to undersampling [7,22]. In this experiment, the S-curve error is clearly displayed and is found only in the gray scale centroid methods. There is no significant S-curve error in the centroiding error of the fitting methods.

#### 3.3.2. Experiment with Different Gaussian Radius

**Figure 5:**
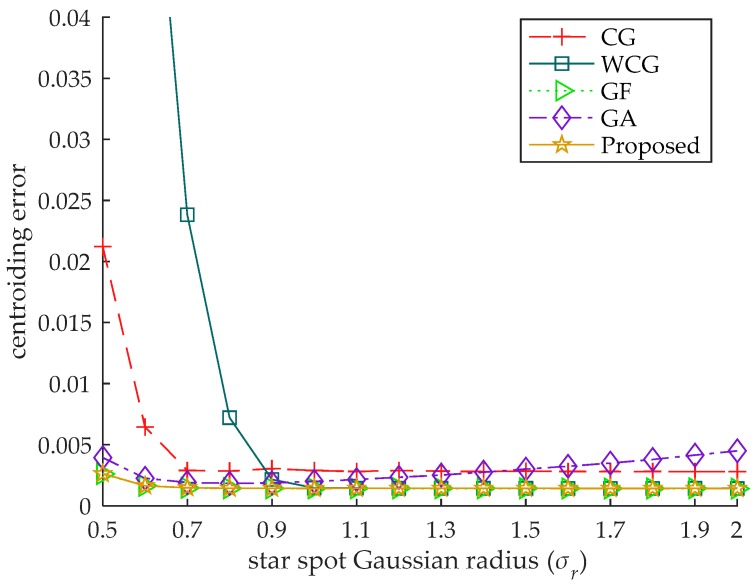
The centroiding error under different spot radius. The centroiding error of the proposed algorithm is almost the same as that of the GF under all the radius of the star spot, less than that of other algorithms.

In this experiment, the accuracy of the algorithm is tested by changing the Gaussian radius of the star spot in the simulated images. The brightness level of each star spot is fixed at 0.7 and the Gaussian radius varies from 0.5 to 2. Gaussian noise with $\sigma_{g}=0.001$ is also added to each image. The centroid of the star spot is uniformly distributed in central pixel of the star image. For each radius, 10,000 simulated star images are generated, and the mean centroiding error of the 10,000 images is used as the centroiding error. The error curves are shown in Figure 5.

Due to the existence of the S-curve error, the centroiding errors of the CG and the WCG are much greater than that of other algorithms when the Gaussian radius is 0.5. As the S-curve error decreases with the increase of the Gaussian radius [7], the centroiding errors of the CG and the WCG first decrease and then tend to be stable. When the error curve is stable, the random noise is the main component of the centroiding error, and the centroiding error of the WCG is less than that of the CG.

As the centroiding errors of the GA, the GF and the proposed algorithm are mainly composed of the random error, the error curves of those algorithms are more stable as shown in Figure 5. When the Gaussian radius is less than 0.6, the random error increases with the decrease of the pixels in the star spot, which leads to the increase of the centroiding error of the GA, the GF and the proposed algorithm.

The GA performs better than the CG and the WCG when the radius of the star spot is relatively small, but its centroiding error is greater than other algorithms when the Gaussian radius of the star spot is greater than 1.4. The centroiding error of the proposed algorithm is almost the same as that of the GF under all the radius of the star spot, less than that of other algorithms.

#### 3.3.3. Experiment with Different Noise Level

**Figure 6:**
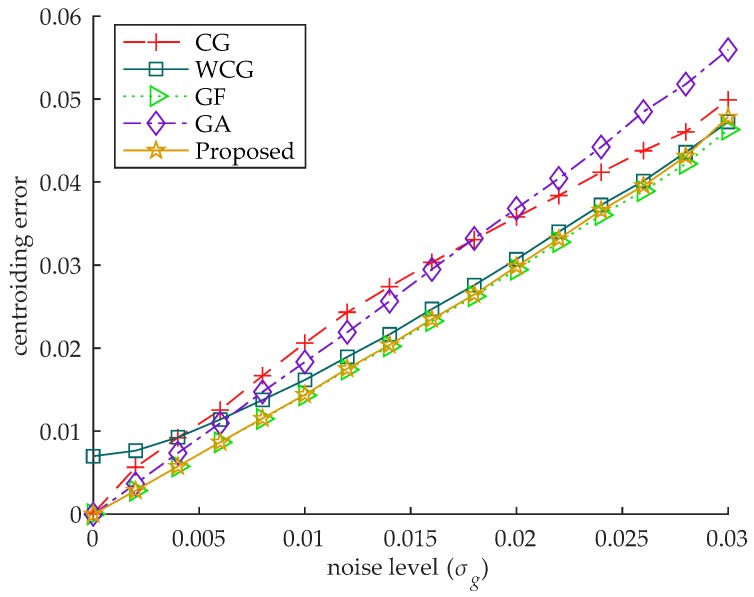
The centroiding error under different level of the noise. The centroiding error of the proposed algorithm is almost the same as that of the GF, less than that of other algorithms under all levels of the noise.

In this experiment, the accuracy of the algorithm is verified by changing the level of the noise added in the star images. The standard deviation of the noise ranges from 0 to 0.03. The Gaussian radius of the star spot is uniformly distributed between 0.5 and 1.2. The brightness level is 0.7. The centroid of the star spot is uniformly distributed within the central pixel of the star image. For each level of the noise, 10,000 images are generated, and the mean centroiding error of the 10,000 images is used as the centroiding error. The error curves are shown in Figure 6.

As seen in Figure 6, with the increase of the noise level, the centroiding error of each algorithm increases gradually. When the noise level $\sigma_{g}$ is greater than 0.006, the centroiding errors of the CG and the GA are similar, both greater than that of other algorithms, indicating that the two algorithms are less robust to noise. The WCG performs better than the CG and the GA when the noise level $\sigma_{g}$ is relatively high, but its centroiding error is the worst when the noise level $\sigma_{g}$ is less than 0.004.

The centroiding error of the proposed algorithm is almost the same as that of the GF, less than other algorithms under all levels of the noise. It indicates that the robustness to noise of the proposed algorithm is almost the same as that of the GF, better than other star centroiding algorithms.

#### 3.3.4. Experiment with Different Brightness Level

**Figure 7:**
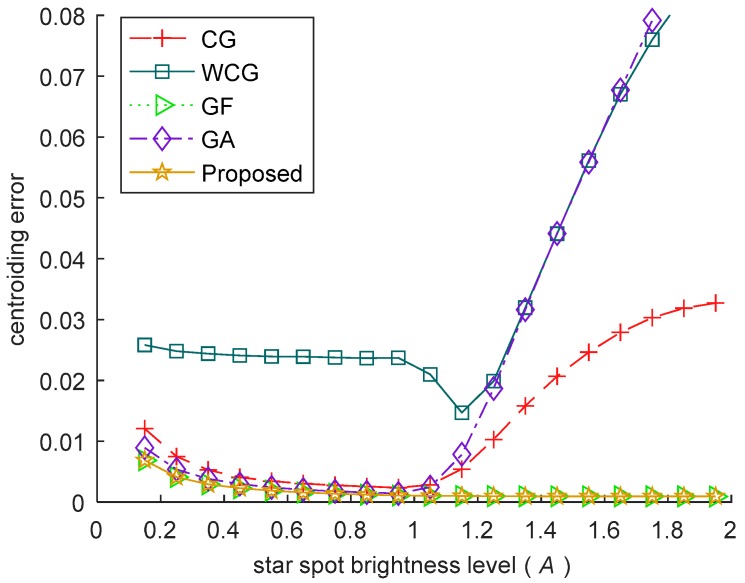
The centroiding error under different brightness level of the star spot. The accuracy of the proposed algorithm is almost stable under all the brightness levels.

In this experiment, the accuracy of the algorithm is verified by changing the brightness level of the stars in the images. The brightness level ranges from 0.15 to 2 (The saturation brightness of each pixel is 1). The Gaussian radius of the star spot is 0.7. The centroid of the star spot is uniformly distributed within the central pixel of the star image. The Gaussian noise with $\sigma_{g}=0.001$ is added to each image. For each level of the brightness, 10,000 images are generated, and the mean centroiding error of the 10,000 images is used as the centroiding error. The error curves are shown in Figure 7.

With the increase of the brightness, there are two main factors affecting the centroiding accuracy:

(i) When a pixel in the star spot is unsaturated, the SNR of the pixel increases with the star getting brighter, helping to improve the accuracy of the algorithm.
(ii) When a pixel is saturated, the gray value of the pixel is truncated, which makes it different from the true value, leading to the increase of the centroiding error.

In Figure 7, factor (i) is the main factor when A≤1, the centroiding error of all the algorithms in this phase reduce because of the increase of SNR. When A continues to get larger, factor (ii) becomes the main factor, the centroiding errors of the CG, the WCG and the GA increase seriously because of the truncation of the saturated pixels. In contrast, the centroiding error of the proposed algorithm and the GF keep stable in this phase. The robustness of the two algorithms results from that the pixels with saturated gray value in the star spot are excluded, as described in Section 2.4.

### 3.4. Efficiency Experiment

The efficiency of the algorithm is also tested and compared with the other algorithms. The total time consumption of each algorithm on 10,000 star images is counted and compared, and listed in Table 1. The brightness level is 0.7 and the Gaussian radius of each star spot is uniformly distributed between 0.5 and 1.2, and the position of the star spot is uniformly distributed within the central pixel of the star image and the standard deviation of the noise is randomly obtained from 0 to 0.02.

There is no doubt that the CG is the fastest. The GF, which takes about 44 times as much time as the CG is the slowest. The WCG and the GA are slightly slower than the CG. The proposed algorithm takes about 3 times the amount of time of the CG, and is about 15 times faster than the GF.

### 3.5. Experiment with Star Sensor Imagery

To further verify the performance of the proposed algorithm, both the CG and the proposed algorithm are implemented and tested on an existing star sensor. Because the true value of the star centroid cannot be obtained, the accuracy of the algorithm is difficult to evaluate. In this section, repeatability is used to evaluate the performance of those two algorithms, and an experiment is performed utilizing a starlight simulator in laboratory.

The star sensor indoor testing system contains a starlight simulator, a two-axis turntable and a tested star sensor. The starlight simulator provides highly collimated starlight with different magnitude, and the turntable provides highly precision test position by rotating its internal frame and external frame. The tested star sensor is mounted on the internal frame of the turntable with its boresight pointing at the starlight simulator. The experiment is carried in a dark room.

By rotating the internal frame and the external frame of the turntable, the star is imaged at different positions of the image plane. For each position, 100 star images are captured and processed by both the two algorithms.

**Figure 8:**
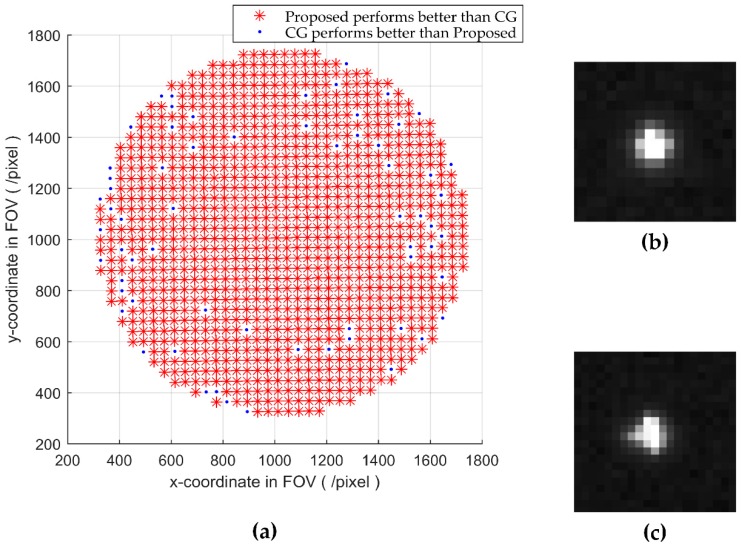
The centroiding result of the two algorithms on an existing star sensor. (**a**) is the positions of all stars, where red asterisks represent the proposed algorithm performs better than that of the CG here; (**b**) is the star spot captured in (1048,847); (**c**) is the star spot captured in (493,559).

As the real position of the star in the image plane cannot be obtained, the performance of the two algorithms is compared according to the standard deviation of the centroids at each position. The algorithm with smaller standard deviation has better repeatability. The comparison results of all the positions are shown in Figure 8.

The points in Figure 8a represent the positions of the stars captured in experiment. The points marked with red asterisks indicate that the proposed algorithm performs better than the CG there, while the points marked with blue dots indicate that the CG performs better. It can be seen that the proposed algorithm performs better at most of the positions. The positions that the CG performs better locate mainly near the edge of the FOV. One typical sTabletar spot at these positions is shown in Figure 8c. Due to the distortion of the optical system, the intensity of the star spot does not conform to Gaussian function strictly, making the proposed algorithm perform slightly worse than CG sometimes. Figure 8b is a star spot located near the center of the FOV, the intensity of which conforms to the Gaussian function better. Table 2 shows the standard deviation of the star spots in Figure 8b,c.

## 4. Conclusions

In order to improve the accuracy of a star sensor, this work focuses on improving the accuracy of the sensor. The Gaussian fitting algorithm is a star centroiding method with high accuracy and low efficiency, and the star centroiding algorithm we proposed aims to improve the efficiency of the Gaussian fitting algorithm without reducing the accuracy. For star images with different centroid, radius, brightness level and noise level, the proposed algorithm performs better than the existing star centroiding methods in terms of accuracy, robustness and efficiency. The proposed algorithm is also evaluated on an existing star sensor, and the results indicate that its repeatability is better than the center of gravity algorithm in most cases. The accuracy and efficiency of this algorithm make it suitable for real-time applications.

## Figures and Tables

**Table 1:** The total time consumption of each algorithm on 10,000 star images.

Method	CG	WCG	GF	GA	Proposed
time (s)	1.3504	1.3878	58.9863	1.4915	3.9675

**Table 2:** The standard deviation of two star spots.

	(1048, 847)		(493, 559)	
	X-Coordinate	Y-Coordinate	X-Coordinate	Y-Coordinate
CG	0.0082	0.0095	0.0159	0.0200
Proposed	0.0060	0.0062	0.0175	0.0233

## References

[1] Liebe C.C. (2002). Accuracy Performance of Star Trackers-A Tutorial. IEEE Trans. Aerosp. Electron. Syst..

[2] Danescu R., Ciurte A., Turcu V. (2014). A low cost automatic detection and ranging system for space surveillance in the medium Earth orbit region and beyond. Sensors.

[3] Hui F., Zhao T., Li X. (2017). Satellite-Based Sea Ice Navigation for Prydz Bay. East Antarctica. Remote Sens..

[4] Vedder J.D. (2015). Star trackers, star catalogs, and attitude determination-Probabilistic aspects of system design. J. Guid. Control Dyn..

[5] Zhang B., Zerubia J., Olivomarin J.C. (2007). Gaussian approximations of fluorescence microscope point-spread function models. Appl. Opt..

[6] Stone R.C. (1989). A comparison of digital centering algorithms. Astron. J..

[7] Wei X., Xu J., Li J. (2014). S-curve centroiding error correction for star sensor. Acta Astronaut..

[8] Rufino G., Accardo D. (2003). Enhancement of the centroiding algorithm for star tracker measure refinement. Acta Astronaut..

[9] Akondi V., Roopashree M.B. Improved iteratively weighted centroiding for accurate spot detection in laser guide star based Shack Hartmann sensor. Proceedings of the Atmospheric and Oceanic Propagation of Electromagnetic Waves IV.

[10] Shortis M.R., Clarke T.A., Short T. Comparison of some techniques for the subpixel location of discrete target images. Proceedings of the Videometrics III.

[11] Lee H.W., Park H.J., Lee J.H. (2007). Accuracy improvement in peak positioning of spectrally distorted fiber Bragg grating sensors by Gaussian curve fitting. Appl. Opt..

[12] Duan Y., Jing P., Niu Z. (2016). Star smear removal for full-frame charge-coupled device images based on Gaussian fitting. J. Appl. Remote Sens..

[13] Jiang J., Xiong K., Yu W. (2016). Star centroiding error compensation for intensified star sensors. Opt. Express.

[14] Wang H., Xu E., Li Z. (2015). Gaussian Analytic Centroiding method of star image of star tracker. Adv. Space Res..

[15] Delabie T., Schutter J.D., Vandenbussche B. (2014). An Accurate and Efficient Gaussian Fit Centroiding Algorithm for Star Trackers. J. Astronaut. Sci..

[16] Flewelling B.R., Mortari D. (2011). Information Theoretic Weighting for Robust Star Centroiding. J. Astronaut. Sci..

[17] Holst G.C. (1998). CCD Arrays, Cameras, and Displays.

[18] Moré J.J., Watson G.A. (1978). The Levenberg-Marquardt algorithm: Implementation and theory. Numerical Analysis.

[19] Coleman T.F., Li Y. (1993). An Interior Trust Region Approach for Nonlinear Minimization Subject to Bounds. SIAM J. Optim..

[20] Shawash J., Selviah D.R. (2012). Real-Time Nonlinear Parameter Estimation Using the Levenberg–Marquardt Algorithm on Field Programmable Gate Arrays. IEEE Trans. Ind. Electron..

[21] Nocedal J., Wright S.J. (2006). Numerical Optimization.

[22] Duren R.M., Liebe C.C. The SRTM sub-arcsecond metrology camera. Proceedings of the 2001 IEEE Aerospace Conference Proceedings.

